# Intra-Uterine Variola

**Published:** 1866-02

**Authors:** 


INTRA-UTERINE VARIOLA.—M. Legros presented to the Biological Society of Paris a variolous fœtus, with the following history; On May 18th, a woman in the Hotel Dieu was prematurely delivered of a fœtus aged apparently about five months, which was covered with pustules of small-pox. The mother had distinct marks of vaccination, and had never had small-pox. About six months previously, she had had connection with a man who was convalescent from variola. No exposure of the mother to contagion could be traced. This case, M. Legros observed, raised the question whether the father could have communicated the small-pox at the moment of fecundation, the disease remaining for five months in a state of latency. This theory he believes to be supported by the facts that, when a pregnant woman has small-pox, the fœtus is sometimes not attacked till some time after the recovery of the mother; and that, in a child of a syphilitic father, the disease in some cases does not show itself in the infant until several days or even weeks after birth.—*Gazette Med. de Paris, Aug.* 5, 1865.

